# New Achievements in High-Pressure Processing to Preserve Human Milk Bioactivity

**DOI:** 10.3389/fped.2018.00323

**Published:** 2018-11-16

**Authors:** Aleksandra Wesolowska, Elena Sinkiewicz-Darol, Olga Barbarska, Kamila Strom, Malgorzata Rutkowska, Katarzyna Karzel, Elzbieta Rosiak, Gabriela Oledzka, Magdalena Orczyk-Pawiłowicz, Sylwester Rzoska, Maria Katarzyna Borszewska-Kornacka

**Affiliations:** ^1^Laboratory of Human Milk and Lactation Research at Regional Human Milk Bank in Holy Family Hospital, Department of Neonatology, Medical University of Warsaw, Warsaw, Poland; ^2^Human Milk Bank, Ludwik Rydygier' Provincial Polyclinical Hospital in Torun, Torun, Poland; ^3^Department of Medical Biology, Medical University of Warsaw, Warsaw, Poland; ^4^First Department of Obstetrics and Gynecology, Medical University of Warsaw, Warsaw, Poland; ^5^High Pressure Physics, Polish Academy of Science, Warsaw, Poland; ^6^Faculty of Psychology, Warsaw University, Warsaw, Poland; ^7^Department of Food Hygiene and Quality Management, Faculty of Human Nutrition and Consumer Science, Warsaw University of Life Sciences, Warsaw, Poland; ^8^Department of Chemistry and Immunochemistry, Wroclaw Medical University, Wroclaw, Poland; ^9^Neonatal and Intensive Care Department, University Hospital, Medical University of Warsaw, Warsaw, Poland

**Keywords:** donor milk, high-pressure processing, milk bank, preterm, adipokines, HGF

## Abstract

High-pressure processing (HPP) is a non-thermal technology that is being increasingly applied in food industries worldwide. It was proposed that this method could be used as an alternative to holder pasteurization (HoP; 62.5°C, 30 min) in milk banks but its impact on the immunologic, enzymatic and hormonal components of human milk has not yet been evaluated in detail. The aim of our study was to compare the effects of HPP in variants: (1) 600 MPa, 10 min (2) 100 MPa, 10 min, interval 10 min, 600 MPa, 10 min (3) 200 MPa, 10 min, interval 10 min, 400 MPa, 10 min (4) 200 MPa, 10 min, interval 10 min, 600 MPa, 10 min in temperature range 19–21°C and HoP on the leptin, adiponectin, insulin, hepatocyte growth factor (HGF), lactoferrin and IgG contents in human milk. HoP was done at the Regional Human Milk Bank in Warsaw at the Holy Family Hospital on S90 Eco pasteurizer (Sterifeed, Medicare Colgate Ltd). Apparatus U4000/65 (Unipress Equipment, Poland) was used for pascalization. Milk samples were obtained from women during 2–6 weeks of lactation. Post-treatment culture showed no endogenous bacterial contamination in any tested option. Concentrations of selected components were determined using ELISA tests. The level of all analyzed components were significantly decreased by HoP: leptin 77.86%, adiponectin 32.79%, insulin 32.40%, HGF 88.72%, lactoferrin 60.31@.%, IgG 49.04%. All HPP variants caused an increase in leptin concentration, respectively (1) 81.79% (2) 90.01% (3) 86.12% (4) 47.96%. Retention of insulin after HPP was (1) 88.20% (2) 81.98% (3) 94.76% (4) 90.31% HGF (1) 36.15% (2) 38.81% 97.15% (3) 97.15% (4) 43.02%, lactoferrin (1) 55.78% (2) 57.63% (3) 78.77% (4) 64.75%. Moreover, HPP variant as 200 + 400 MPa preserved IgG (82.24%) better than HoP and resulted not statistically significant change of adiponectin level (38.55%) compare to raw milk. Our results showed that HPP leads to preservation of adipokines, growth factor, and lactoferrin, IgG much better or comparable with HoP.

## Introduction

Mother's milk is a natural first choice feed for every newborn, whether born in term or prematurely. Access to human milk is critical especially for very preterm babies for their current health condition and later life prognosis (1, 2). In those circumstances, human milk has a not only nutritional function but is a source of non-nutritive bioactive compounds. The presumably, cumulative effects of thousands of substance such as anti-inflammatory agents, immunoglobulins, cytokines, growth factors, oligosaccharides, and bioactive peptides from human milk exist in preventing serious complications of prematurity such as necrotizing enterocolitis (NEC) (3–7). Many of these substances such as hormones and cytokines, even have potential for long –term metabolic programming (8).

Hormones as insulin, leptin, adiponectin have impact on infant growth and body composition. **H**epatocyte **G**rowth **F**actor (HGF) and multifunctional milk protein as lactoferrin act in synergy to support the function of the immature gastrointestinal tract of newborns (5, 9–13). Current knowledge about benefit of bioactive factors in breastmilk for infants in early life has results in the increasing number of human milk banks. Therefore, when mother's own milk is unavailable, donor milk is recommended (2, 14). Nearly 80 new units located mostly in hospitals specialized in tertiary neonatal centers and NICU were organized in the last decade in European countries alone (15–17). These professional laboratories operate by screening, collecting, processing, and distributing human milk that has been donated by volunteer nursing mothers unrelated to the recipient infant (18). Although human milk banks are well-equipped and organized, the processing of donor milk needs improvements due to the partial loss of its bioactive properties compared with raw milk (19).

Pasteurization used in mostly human milk banks for microbiological purity consists of heating to 62.5°C for 30 min with obvious side effects for many human milk constituents (20). This approach is especially harmful for non-nutritive elements of human milk such as enzymes, hormones, growth factors and cytokines, leading to their diminished presence and activity. Given this side effect of holder pasteurization, new techniques of processing donated human milk are needed to preserve its bioactivity (19, 21).

In the present research bioactivity of several components of human milk after standard and innovative high pressure processing were evaluated.

## Materials and methods

### Samples collection

Milk samples were obtained from 80 donors of the Regional Human Milk Bank in Warsaw at Holy Family Hospital. Donors were given written and verbal instructions on expressing and handling of milk and cleaning of breast pumps. Milk samples were obtained from women (average age was 31 year old) after delivery at term with a surplus of milk during the 2–6 weeks of lactation given informed consent. Samples of ~50 ml of milk were collected at home or in the hospital ward using an electric or manual pump, stored in a refrigerator at temperature 4°C and delivered to human milk bank unit within 24 h while maintaining the refrigeration conditions.

The Bioethics Committee of Warsaw Medical University has accepted the information about conducting this non–interventional study (admission number AKBE/59/15).

### Experimental design and samples preparation

**Figure 1:**
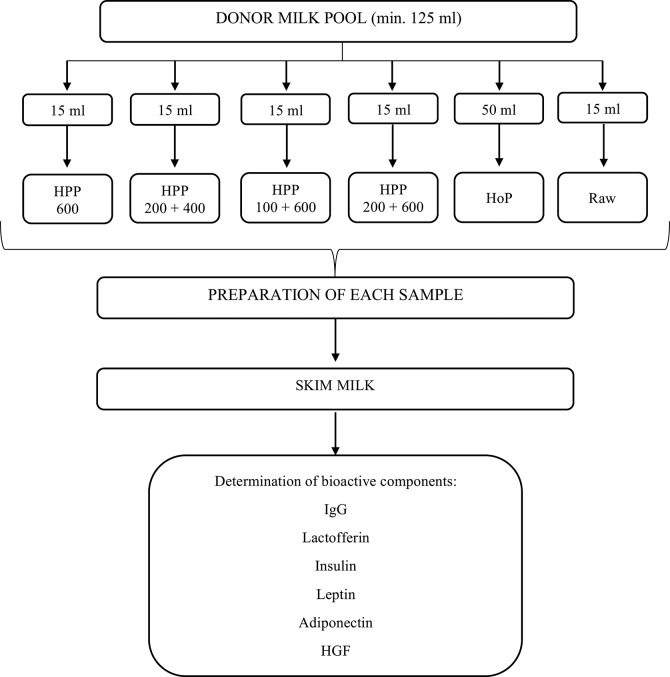
Experimental design of the study.

The same volume of milk samples from 2 to 4 donors were pooled to achieve the minimum volume of minimum 125 ml necessary for the study. Each batch was divided into aliquots and exposed to 4 variants of **H**igh **P**ressure **P**rocessing (HPP): (1) 600 MPa, (2) 200 + 400 MPa, (3) 100 + 600 MPa, (4) 200 + 600 MPa and holder pasteurization (HoP). The control sample was raw, untreated human milk (Figure 1). The experiments were made three times on independent milk batch.

Following the HPP and HoP treatment all samples as well as raw untreated milk were centrifuged at 4,400 rpm for 15 min at 4°C (Centrifuge 5702R, Eppendorf) after which the fat layer and cells were removed and supernatants were aliquoted into Eppendorf tubes prior to freezing at −21°C. Human milk samples were frozen within 48 h of collection.

Microbiological analysis were performed to verify microbiological purity in the case of selected raw and treated milk samples. The analysis were carried out in three replications to the total number of mesophilic aerobic microorganisms (PN-EN ISO 7218: 2008 / A1: 2013, PN-EN ISO 6887-5: 2010) and the number and *Staphylococcus aureus* (PN-EN ISO 6888-1: 2001 / A1: 2004).

### Treatment

#### High pressure processing

Human milk samples were exposed to high pressure treatment at the Institute of High Pressure Physics, Polish Academy of Sciences, using U 4000/65 apparatus (designed and produced by Unipress Equipment). The maximum pressure available in the apparatus was 600 MPa, the treatment chamber had a volume of 0.95 L. The pressure-transmitting fluid used was distilled water and polypropylene glycol (1:1). Manufactory designed working temperature of the apparatus ranges from −10°C to +80°C. In our experiments, the temperature of the tested condition was between 19 and 21°C. A pressure of up to 600 MPa was generated in 15–25 s; the release time was 1–4 s.19 and 21°C. Samples were prepared in 4 variants: (1) 600 MPa (2) 200 MPa, 10 min; interval 10 min; 400 MPa, 10 min (3) 100 MPa, 10 min; interval 10 min; 600 MPa, 10 min (4) 200 MPa, 10 min; interval, 10 min; 600 MPa, 10 min.

#### Holder pasteurization

Holder Pasteurization (HoP) of human milk samples was done at the Regional Human Milk Bank in Warsaw at the Holy Family Hospital on automatic Human Milk Pasteuriser S90 Eco (Sterifeed, Medicare Colgate Ltd). Samples of 50 ml were treated according to Regional Human Milk Bank standard pasteurization protocol at 62.5°C for 30 min. The correctness of the process was confirmed with the data logging system, by recording temperature of the bottle probe every minute.

### Determination of bioactive components

All of the bioactive components were determined by the ELISA method. The assay detecting particular components in the milk was done at least in three times using milk samples proccesing in independent experiments. The concentration of IgG was determined according to a procedure described earlier (22). Briefly: the F(ab')_2_ fragment of goat anti-human IgG (Jackson ImmunoResearch, USA) was used as a coating agent of the wells of a microtiter plate (Nalge Nunc International, Naperville, IL, USA) to bind IgG from the sample. For testing 100 μl of 100 μl of 100-, 250-, 500-, and 1,000-fold diluted milk and IgG standard preparation from 0.2 to 12.5 ng/100 μl (Jackson ImmunoResearch, USA) were taken. The amount of IgG bound was quantified by phosphatase-labeled rabbit anti-human IgG Fcγ fragment specific antibodies (Jackson ImmunoResearch, USA).

For lactoferrin determination monoclonal anti-human lactoferrin antibody (ABCAM, Cambridge, UK) was used as a coating agent of the wells of a microtiter plate (Nalge Nunc International, Naperville, IL, USA) to bind lactoferrin from the sample. For testing 100 μl of 5 000-, 10 000-, 25 000- and 50 000- fold diluted milk and lactoferrin standard preparation from 0.8 to 25 ng/100 μl (Sigma, St. Louis, MO, USA) were taken. The amount of lactoferrin bound was quantified by phosphatase-labeled rabbit anti-human lactoferrin antibodies (Jackson ImmunoResearch, USA).

The IgG and lactoferrin tests were assayed with 4-nitrophenyl phosphate (SERVA, Heidelberg, Germany) as the enzyme substrate and absorbance was measured in a Stat Fax 2100 Microplate Reader (Awareness Technology Inc., Palm City, FL, USA) at 405 nm with 630 nm as the reference filter. All ELISA immunobinding and washing steps were carried out in a TRIS-buffered saline (TBS, pH 7.5) containing 0.2% Tween 20. All samples were analyzed at four different sample dilutions, each in duplicate.

The concentrations of leptin, adiponectin, HGF, and insulin were analyzed with commercial ELISA kits using microplates pre-coated with a monoclonal antibody specifically for test substances. The following tests were used: Human Leptin (R&D Systems, Inc); Human HMW Adiponectin/Acrp30 (R&D Systems, Inc); Human HGF (R&D Systems, Inc); Insulin ELISA (DRG Instruments GmbH, Germany). For the study, the option for serum/plasma was chosen as the most adequate for human milk. The method was pre-tested on various samples dilutions as proposed in the protocol. As a result of this validation, the final analyses were performed on undiluted milk. Each sample in ELISA assay was measured in duplicate.

The detections of adiponectin, HGF and leptin, insulin were done by microtiter reader (Synergy HTX multimode reader, Biotek®) set to 450 nm with 570 nm as the reference filter. For data analysis Gen5 Data Analysis Software was used.

### Statistics

Statistical analysis concerns results obtained for six conditions: raw milk, pasteurized milk (HoP), and milk exposed to four variants of high pressure processing (HPP): 600 MPa, 200 MPa +400 MPa, 100 MPa+ 600 MPa, 200 MPa+ 600 MPa. As a calculation tool MS Excel was used. The results of determined parameters for each sample are showed as a percentage of the initial value observed for raw milk as 100%. Next, means, SD and 95% coefficient intervals (95% CI, which refers to *p* ≤ 0.05) were computed. The actual analysis was based on the overview of overlapping of obtained intervals.

## Results

The average value of the total viable number of microorganisms in raw milk was determined on the level 3.3 ± 0.90 log cfu / ml. The number of *S. aureus* was determined at 1.57 ± 0.65 log cfu/ml.

Microbiological analyses carried out in pasteurized and pascalised human milk samples did not show the presence of the selected microorganisms (Supplementary Table 1).

The concentration of bioactive components in raw milk samples are shown in Table 1.

**Table 1:** Concentration of bioactive components in raw milk.

	**IgG (μg/ml)**	**Lactoferrin (mg/ml)**	**Leptin (pg/ml)**	**Adiponectin (ng/ml)**	**HGF (mlU/ml)**	**Insulin (pg/ml)**
Mean ± SD	11.22 ± 8.83	1.63 ± 0.47	269.97± 56.53	5.30 ± 2.05	1306.15± 956.99	10.24 ± 4.02
Min.	5.74	1.17	226.16	2.94	413.20	6.10
Max.	21.40	2.11	333.78	3.68	2261.00	14.67

*The results are presented as mean ± standard deviation with indication on minimum and maximum*.

**Figure 2:**
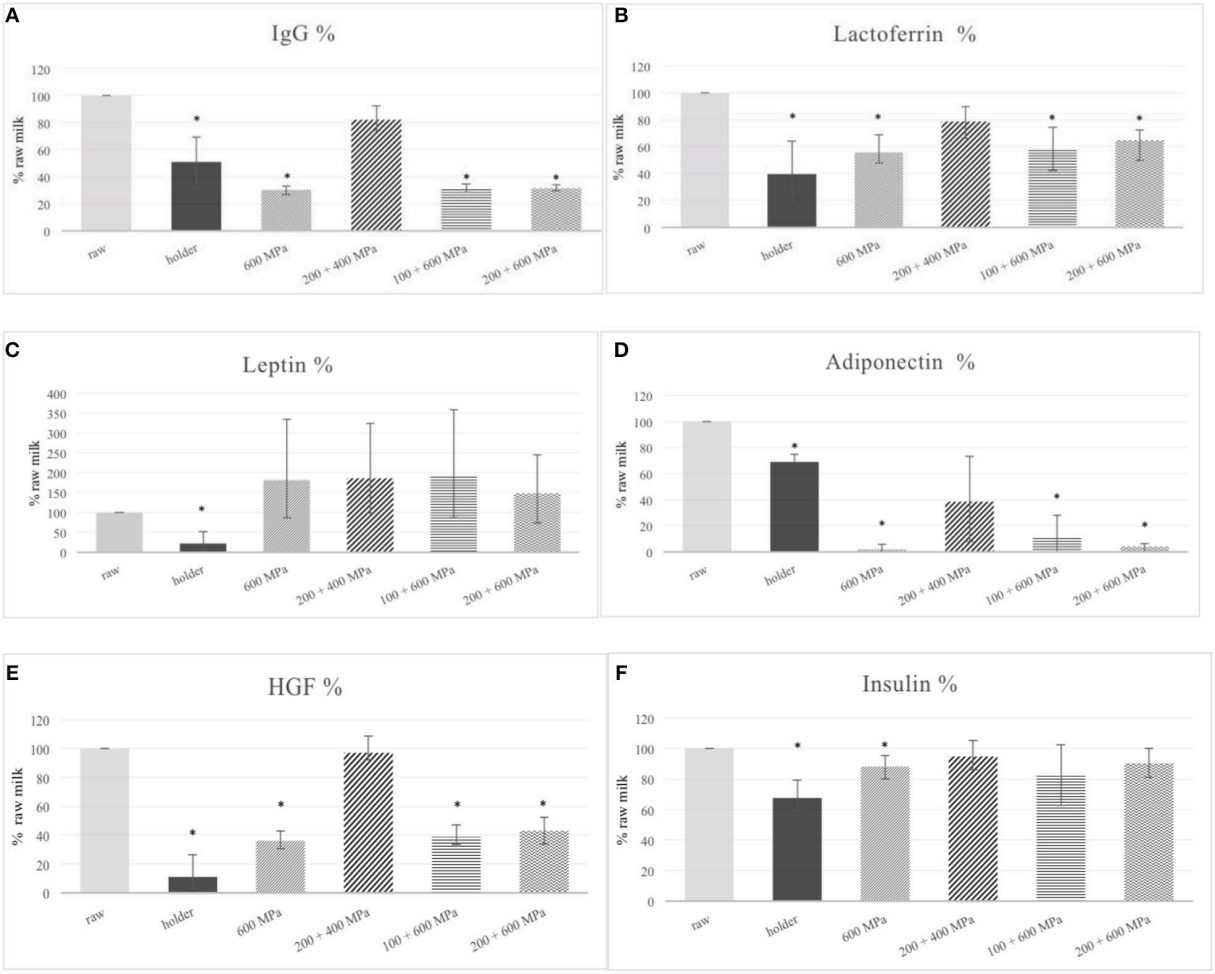
Changes in IgG **(A)**, lactofferin **(B)**, leptin **(C)**, adiponectin **(D)**, HGF **(E)**, insulin **(F)** content in human milk after processing. ^*^p ≤ 0.05.

Results of our experiments revealed that HoP caused a statistically significant reduction (49.04%) in IgG content. HPP variants 600 MPa, 100 MPa +600 MPa, and 200 MPa + 600 MPa also decreased statistically significantly the IgG content, 69.68, 69.16, and 68.46%, respectively. The reduction of IgG in 200 MPa +400 MPa (17.76%) was not statistically significant (Table 2, Figure 2A).

**Table 2:** Changes in the content of IgG, lactoferrin leptin, adiponectin, HGF, and insulin in human milk after preserving with different methods.

**Bioactive components**	**Raw milk (%)**	**Holder (%)**	**High-Pressure Processing**			
			**600 MPa (%)**	**100MPa +600 MPa (%)**	**200 MPa +400 MPa (%)**	**200 MPa +600 MPa (%)**
IgG	100	50.96	30.32	30.84	82.24	31.54
Lactoferrin	100	39.69	55.78	57.63	78.77	64.75
Leptin	100	22.14	181.71	190.01	186.12	147.96
Adiponectin	100	67.21	2.01	10.73	38.55	4.09
HGF	100	11.28	36.15	38.81	97.15	43.02
Insulin	100	67.6	88.20	81.98	94.76	90.31

*The results are presented as a retention percentage compare to the content in raw milk (mean values)*.

In the case of lactoferrin HoP caused a statistically significant reduction of this protein (60.31%) in the human milk. HPP variants 600 MPa, 100 MPa +600 MPa, and 200 MPa + 600 MPa also decreased the lactoferrin content statistically significantly, 44.22, 42.37, and 35.25%, respectively. The reduction of lactoferrin in 200 MPa +400 MPa (21.23%) was not statistically significant (Table 2, Figure 2B).

Leptin level was significantly reduced by HoP (77.86%) in comparison to the raw milk. In the matter of high—pressure, all HPP variants caused an increase in leptin concentration, (1) 600 MPa; 81.71%, (2) 200 MPa +400 MPa; 86.12%, (3) 100 MPa + 600 MPa; 90.01%, (4) 200 MPa +600 MPa; 47.96%, respectively. Indeed, none of the high pressure variants were significantly different from raw milk (Figure 2C).

HoP caused a statistically significant decrease in adiponectin content (31.19%) but not so serious as HPP variants 600 MPa, 100 MPa +600 MPa, and 200 MPa + 600 MPa which reduced the level of protein almost totally: respectively as 97.99, 89.27, and 95.91%. The reduction of adiponectin at 200 MPa +400 MPa was slighter (61.45%), but also statistically significant (Figure 2D).

Results obtained for HGF were very similar to adiponectin. HoP caused a statistically significant reduction level of HGF detected in human milk (88.72%). Although the level after HPP treatment by 600 MPa, 100 MPa +600 MPa, and 200 MPa + 600 MPa was decreased not more than 63.85, 61.19, and 56.98%, respectively, it was still significant. Only when it comes to HPP variant 200 MPa +400 MPa was the change in HGF level was almost imperceptible and not statistically significant (2.85%) (Figure 2E).

The level of insulin was diminished under the influence of HoP by 32.40% in comparison to raw milk. Among of HPP variants only the treatment of human milk by high pressure as 600 MPa caused statistically significant destruction of protein (11.80%). The reduction in insulin content in human milk after others HPP variants was as following: 200 MPa +400 MPa-−5.24%, 100 MPa + 600 MPa-−18.02%, 200 MPa +600 Mpa-−9.69% but it was not statistically significant (Figure 2F).

The content of selected bioactive compounds in raw milk was assumed as 100%, additionally the range of obtained results (minimum-maximum) was presented as an error bar. The asterisks indicate a pair of results that differ statistically significantly with *p* ≤ 0.05.

## Discussion

The human milk donated for human milk banks needs to be of very high quality concerning microbiological safety, nutritional value, and last but not least, bioactivity. For this reason, an operational procedure has been implemented to monitor the whole process of human milk bank activity. National guidelines have been developed in many countries to improve the standards for recruitment, screen the donors and handle and distribute the collected milk (23–25). There are, many minor differences in operational procedure among milk banks in Europe but the pasteurization stage has a common core process. Holder pasteurization has been a “gold standard” in milk banks worldwide for many years. This process involves heating the milk to 62.5°C within 30 min. The relatively low temperature and long time parameters (for this reason called also **L**ow **T**emperature **L**ong **T**ime pasteurization, LTLT) was combined to assure microbiological safety and nutritional value (26). Ever since this technique was incorporated into the milk bank system, it has been known to be damaging for many bioactive milk components, such as vitamins: C, folacin, and B6, poly-unsaturated fatty acids and free fatty acid composition (27–30).

The current knowledge of this topic is summarized in a systematic review by Peila et al. (20). The authors distinguished three groups of human milk components according to the influence from holder pasteurization: those significantly affected such as: enzymes, some cytokines, growth factors: IGF, EPO, HGF, GM-GSF, hormones: insulin, adiponectin, vitamins: B6, ascorbic acid, antioxidant capacity, content of nucleotide monophosphate, free amino acid; Those affected but with contradictory results: immunoglobulins S-IgA, IgM, IgG, lactofferin, lysozyme, some cytokines, some vitamins, total fat content including, saturated fatty acid, —mono and polyunsaturated fatty acid. Fortunately there are some thermal resistance components found in human milk such as some cytokines and growth factors, amino acid, some vitamins: D, E, B2, B5, biotin, B3, B12, antioxidant capacity, lipids, total nitrogen content, human milk oligosaccharides. The great heterogeneity in the available data is partially due to a lack of standardized study protocols in this fields. However, the reduced value of pasteurization is great enough to take immediate steps in searching for a technical solution for milk banks.

HPP is one of the most promising alternatives for thermal treatment, but this sophisticated technique has not yet been validated for human milk. For our study we have chosen those components of human milk that were known to be affected by holder pasteurization but hadn't been evaluated in concern on HPP. We focused on biologically active peptides represented by adiponectin, insulin, leptin and HGF because of its impact on metabolism regulation in newborn. In fact the available data concerning preservation of those human milk components are sparing in details. We could only find one study showing the destruction of insulin and adiponectin under the influence of a standard holder treatment (31). However in case of leptin the work has been done on fast—and slow-heat pasteurization 100°C in 5 min and 57°C in 30 min, respectively (32). In all these cases, it was observed that the active peptin level was diminished in the range of 30–40% compared to unpasteurized milk.

Moreover, we included in our research two immunologically important proteins from human milk such as immunoglobulin G (IgG) and lactoferrin. Preservation on immunocomponents in donor milk, are well documented in spite of contradictory results (33–38). Nevertheless, we used those two components as an indicator to compare with trends observed for biologically active peptides of non-nutrition value after thermal and high pressure treated milk.

In our study the mean IgG concentration in untreated milk samples was 11.22 ± 8.83 μg/ml (range 5.74–21.40 μg/ml) Table 1. IgG is reported as a very sensitive immunoglobulin with IG4 subclass which was thermal resistance. The loss in the case of holder pasteurization is about 50% relative to untreated milk. Our results are consistent with others, reported by Sousa et al. (38). Only one of the HPP variants (200 MPa +400 MPa) did not statistically significantly decrease the IgG content in milk samples (reduction 17.76%) and gave results better than holder pasteurization (Figure 2A).

In the case of lactoferrin the mean concentration detected in raw milk samples was 1.63 ± 0.47 mg/ml (range 1.17–2.11 mg/ml) which is similar to those reported in the literature (39) (Table 1) The reduction of lactoferrin level by HoP in current experiments is 60% of the level detected in raw milk samples. The difference was statistically significant. It is in the range from 35 to 90% losses observed by others researches. Still it is twice more preserve than was reported by Christen et al. as a 20% retention after HoP (36). All HPP variants used in this study better preserved lactoferrin in human milk than holder pasteurization. Because of the great variance of data the difference was not statistically significant. However, we could see that the best option of HPP is 200 MPa +400 MPa variant, which resulted only 21.23% diminished of lactoferrin level which was not statistically significant comparing to raw milk (Figure 2B).

Taking into account the importance of short and long lasting outcome of donor milk nutritional therapy, we decided to extend the research in to evaluate the possibility to preserve the biologically active peptides found in human milk as key metabolism regulation components.

We suspected that HPP variant 200 MPa +400 MPa already tested with success for immunocomponents preservation, will be preferable in comparison to thermal treatment, for hormones such as leptin, adiponectin, insulin, and HGF as well.

As was revealed before, HPP was even most effective as holder pasteurization in the elimination of inoculated microbiological flora of human milk (40). In our current study HPP was able to eliminate commensal bacteria of donors milk successfully (Supplementary Data).

The most recent update of Cochrane metaanalysis which evaluated growth and development of preterm born infants fed with formula comparing with donor milk has proven that supplementing mother's milk with pasteurized human milk results in lower rates of weight gain, linear growth and head growth (41). Although it is more important that diet based on solely human milk reduces the NEC risk, hesitation about the consequence of long term under nutrition remains. In this context it seems to be most important to preservation donor milk components with regulation metabolism properties.

Among adipokines derived from human milk, leptin, and adiponectin, have great impacts on the neonatal growth and development. Leptin is a key factor in the regulation of energy balance and appetite (42, 43). Blood leptin concentration in **S**mall for-**G**estational **A**ge (ang. SGA) neonates has been observed to be inversely related to rates of intrauterine growth, suggesting a possible role of leptin in promoting fetus growth (44). Leptin in human milk appears from mammary epithelial cells in milk fat globules as well as being transferred from material blood (45, 46). Leptin receptors have been identified in the human small intestine, which suggests that breast milk leptin could play a role in the short-term control of food intake in neonates (47).

In our experiments, leptin hormone was detected in all analyzed milk samples before and after processing. The mean concentration of leptin in raw milk samples was 269.97 ± 56.53 pg/ml (range 226.16–333.78 pg/ml) which is similar to those reported in the literature (48) (Table 1). Leptin seems to be a thermolabile protein, therefore it is not uncommon that treatment in 62.5°C by 30 min decreases the detectable level more than 70%, more slightly sterilization condition as 57°C caused comparable results, as was shown earlier. Surprisingly, after high pressure processing we even detected an increase relative to untreated milk (Figure 2C, Table 2). This phenomena could be explained by the influence of hydrostatic pressure on the physicochemical property of human milk. Human milk is a very complex biological fluid that could be characterized simply as an emulsion of fat globules in an aqueous liquid with cells components. As was shown leptin is located predominantly in emulsion phase of human milk, which consists of the milk fat droplet or fat-associated proteins. Some portion of human milk leptin is locally synthesized in mammary epithelium cells. In fact documented effects of high pressure on milk lipids have been scarce. However, milk fat globules appear to remain intact under pressure, some alternation in globule size being observed (49). It is not ruled out that pascalization of human milk causes the release of leptin incorporated in milk fat globule or in cellular component of human milk. Indeed, because of high variance, the results after HPP treatment were not statistically significantly different from raw milk.

In the case of adiponectin, the mean concentration detected in raw milk samples was 5.30 ± 2.05 ng/ml (range 2.94–3.68 ng/ml) which was comparable to others findings (Table 1). The average quantity of adiponectin in human milk detected by Martin LJ and coworkers was ~19 ng/ml (range 4–88 ng/ml) (50). In another study median adiponectin concentration in human milk was 9.99 ng/ml (range 3.59–20.52 ng/ml). Adiponectin levels remain well detectable throughout the time of breastfeeding with a high level at the beginning of the course of lactation and with a decrease at the time of the introduction of complementary feeding (51). Adiponectin, synthesized by adipocytes, exists in plasma as a several different oligomeric proteins. High-molecular-weight (HMW) adiponectin is a large multimer of 12–18 subunits, thought to be the most biologically active form almost entirely existing in human milk (9). Therefore, we evaluated this particular form of protein in the human milk after processing. We have detected HMV adiponectin in all analyzed human milk samples and we could observed that all methods of treatment significantly decreased the content of adiponectin in milk samples compared to raw milk (Figure 2D, Table 2). Fortunately, the reduction of adiponectin in HPP variant 200 MPa +400 MPa was not so radical but not very much different from after HoP. As was mentioned earlier, because of the crucial role adiponectin in the regulation of insulin sensitivity in the offspring, reduction of functional hormone in donor milk could be especially problematic for very preterm infants who are at increased risk of insulin resistance and type 2 diabetes later in life (32).

The mean concentration of HGF in raw milk samples was 1306.15 ± 956.99 mlU/ml (range 413.20–2261 mlU/ml) (Table 1). In spite of concentrations of HGF in human milk is dependent on many factors including selection of the study group and demographic variables, but the stage of lactation seems to be crucial (52). The HGF level in colostrum is 20–30 higher than in mature milk, which stresses the key role of this growth factor in the development progress of newborns. Moreover, the concentration of HGF in milk from mothers delivered preterm were significantly higher than those from term deliveries. As was speculated earlier, it was finally discovered that trophic action of HGF from human milk occurs by the stimulation of gastrointestinal (GI) epithelial cells (53, 54). In this context, the presence of HGF in donor milk could represent a special benefit in prevention of such serious GI track illnesses such as NEC. As was n in Figure 2E the HGF level after treatment by HPP 200 MPa +400 MPa did not significantly differ from the level in raw milk but it was significantly higher than the level of HGF in milk subjected to other high pressure variants or HoP.

Insulin has been detected at very high levels in the human colostrum of healthy mothers (114–306 mU/L) after that decreasing by day 5 postpartum to the physiological levels at the fasting state (55). Insulin seems to be actively transported into milk irrespective of the source because exogenous insulin used for treatment of type 1 diabetes is found in human milk. Surprisingly, that levels of insulin are significantly higher in milk from type 1 diabetic mothers than that of non-diabetic control mothers (56). As has been show earlier, insulin derived from derived insulin could work effectively in newborn exerted hormone dependent effect to glucose homeostasis (57). In addition to the key role of insulin in blood glucose the homeostasis, the properties of dietary hormone in influencing growth and development of the small intestine was postulated (58). Recent studies have shown the link of human milk hormone occurrence, as insulin as well as leptin, with the proper pattern of intestinal microbiome in neonates (59).

In our study the mean insulin concentration in raw milk samples was 10.24 ± 4.02 pg/ml (range 6.10–14.67 pg/ml) which is similar to data reported in the literature (60) Table 1. Thermal treatment used in experiments affect significantly the content of insulin in pasteurized human milk compared to raw milk. The pasteurization changes occurred were comparable with the remarkable decreased observed by Ley (32) (Figure 2F). In the case of three out of four HPP variants, including HPP 200 MPa +400 MPa, they gave results significantly better than holder pasteurization.

In conclusion, our experiments showed that the 200+400 MPa variant is the best option of high pressure to preserve several metabolic hormones and immunocomponents of human milk. Our findings with persistent bioactive peptides in pascalized human milk is in line with the earliest reports concerning retention of others hormones after HPP with great significance for the infant's health (21, 61, 62).

We showed, for the first time, preservation of several metabolic hormones in donor milk HPP processing. Growing recent studies in nutritional therapy of preterm's have emphasized the role of our discovery (63, 64).

We believed that preservation of important bioactive peptides as hormones and growth factors in human milk is especially important in the context of delivering donor milk for very preterm infants.

## Limitation of the study and future direction

Lack of protocol for evaluation of new techniques was a severe obstacle to improving the pasteurization stage of donor milk. In the next step we would like to follow current expert's recommendation for validation of new method of human milk processing and fit designed methodology for this, especially in the matter of microbiology.

## Author contributions

AW: main contributions to the conception and design of the work, analysis and interpretation of data for the work, drafting the work. ES-D and OB: acquisition, analysis and interpretation of data for the work, revising it critically for important intellectual content. KS and MR: acquiring the main part of the data used for the work. KK statistical analysis and interpretation of data for the work. ER: acquiring part of the data for the work. GO, SR, and MB-K: revising it critically for important intellectual content, providing approval for publication of the content. MO-P: acquiring part of the data for the work, revising it critically for important intellectual content. All authors agree to be accountable for all aspects of the work in ensuring that questions related to the accuracy or integrity of any part of the work are appropriately investigated and resolved.

### Conflict of interest statement

The authors declare that the research was conducted in the absence of any commercial or financial relationships that could be construed as a potential conflict of interest.
