# Supplementary material for: New Achievements in High-Pressure Processing to Preserve Human Milk Bioactivity
Source: Front Pediatr. 2018 Nov 16;6:323. doi: 10.3389/fped.2018.00323 (PMC6250976; doi:10.3389/fped.2018.00323)
Supplement: Supplementary file 1 [file Table_1.docx]

Supplementary Material

Article Title

**New achievements in high-pressure processing to preserve human milk bioactivity**

Aleksandra Wesolowska* ^1,2^, Elena Sinkiewicz-Darol ^1,2^, Olga Barbarska^1,3,4^ Kamila Strom^3^, Malgorzata Rutkowska^5^, Katarzyna Karzel^6^, Elzbieta Rosiak, ^7^ Gabriela Oledzka^3^, Magdalena Orczyk-Pawiłowicz^8^, Sylwester Rzoska^5^ and Maria Katarzyna Borszewska-Kornacka^9^

1. Laboratory of Human Milk and Lactation Research at Regional Human Milk Bank in Holy Hospital , Medical University of Warsaw, Department of Neonatology, Warsaw, Poland
2. Human Milk Bank, Ludwik Rydygier’ Provincial Polyclinical Hospital in Torun, Torun, Poland
3. Department of Medical Biology, Medical University of Warsaw, Warsaw, Poland;
4. Medical University of Warsaw, First Department of Obstetrics and Gynecology, Warsaw, Poland;
5. High Pressure Physics, Polish Academy of Science, Warsaw, Poland;
6. Warsaw University, Faculty of Psychology, Warsaw University, Warsaw, Poland;
7. Warsaw University of Life Sciences, Faculty of Human Nutrition and Consumer Sciences, Department of Food Hygiene and Quality Management, Warsaw, Poland
8. Department of Chemistry and Immunochemistry, Wroclaw Medical University, Wroclaw, Poland
9. University Hospital, Neonatal and Intensive Care Department, Medical University of Warsaw, Warsaw, Poland

* Correspondence author

Aleksandra Wesolowska

[aleksandara.wesolowska@wum.edu.pl](mailto:aleksandara.wesolowska@wum.edu.pl)

# Supplementary Tables

Table 1 Microbiology analyses of raw milk and after processing

|  | TVMC  [log cfu/ml] | S. aureus  [log cfu/ml] |
| --- | --- | --- |
| Raw milk | 3,3±0,90 | 1,57±0,65 |
| Pasteurized/Pascalised milk | 0 | 0 |
